# Making Living-donor Liver Transplantation a Viable Option for Patients With Portopulmonary Hypertension

**DOI:** 10.1097/TXD.0000000000001710

**Published:** 2024-09-25

**Authors:** Kristen Burton, Andrew Gold, Peter Abt, Nolan Machado, Kristen Rock, Dmitri Bezinover

**Affiliations:** 1 Department of Anesthesiology and Critical Care, Perelman School of Medicine, University of Pennsylvania, Philadelphia, PA.; 2 Department of Surgery, Perelman School of Medicine, University of Pennsylvania, Philadelphia, PA.

## Abstract

Liver transplantation (LT) in patients with significant portopulmonary hypertension (PoPH) is associated with an increased risk of several complications, including graft failure. Graft loss is one of the major reasons. Living donor LT (LDLT) is not routinely performed in the United States in this patient population. In addition, ethical considerations often preclude donation from healthy donors in the setting of a procedure associated with an elevated risk of recipient morbidity and mortality. However, LDLT allows LT to be performed electively, using a superior graft with an improved probability of a good outcome. The key to success in managing these patients is establishing a multidisciplinary team that follows an institutional protocol with clear evaluation and management criteria. These criteria include screening and early diagnosis as well as treatment of PoPH with the goal of optimizing pulmonary arterial hemodynamics and maintaining right ventricular function. Any protocol should include admitting the patient to the hospital a day before surgery for placement of a pulmonary artery catheter to measure and derive relevant hemodynamic variables. A multidisciplinary team should determine the fitness for a transplant a after a careful review of the most up-to-date clinical information. Finally, the team prescribes and executes a plan for optimization and safe perioperative management of the patient. In this report, we discuss our approach to the perioperative management of a patient with significant PoPH who safely underwent LDLT with an excellent postoperative outcome.

## Graphical abstract

**Figure:**
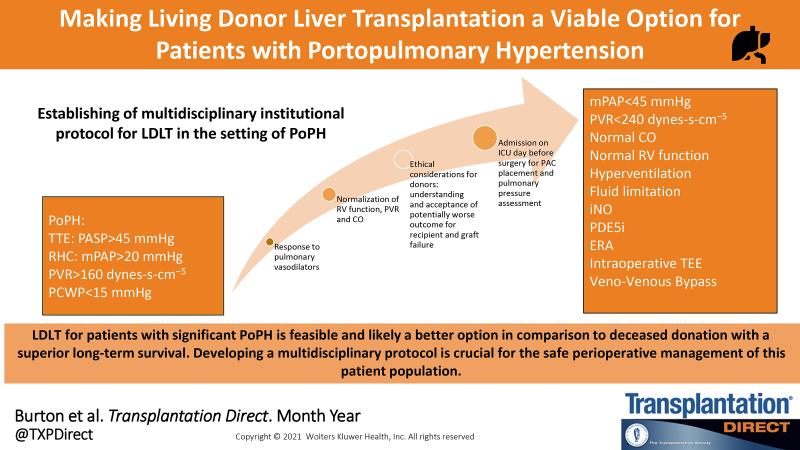

Portopulmonary hypertension (PoPH), a subtype of pulmonary arterial hypertension, presents as a progressive disorder in patients with chronic liver disease (with or without cirrhosis) in the setting of portal hypertension. PoPH is defined by the after RHC hemodynamic parameters: mean pulmonary artery pressure (mPAP) >25 mm Hg, pulmonary vascular resistance (PVR) >240 dynes-s-cm^−5^, and pulmonary capillary wedge pressure (PCWP) <15 mm Hg.^1,2^ In the most recent articles, PVR >160 dynes-s-cm^−5^ was described as one of the most important criteria for diagnosis of PoPH.^3^ PoPH has 3 degrees of severity: mild (mPAP at rest >25 to <35 mm Hg), moderate (mPAP at rest ≥35 to <45 mm Hg), and severe (mPAP at rest ≥45 mm Hg).^4^ The prevalence of PoPH is 2%–10% in patients with portal hypertension,^5,6^ and is associated with high mortality.^1,7^ It has been demonstrated that the symptoms of PoPH can be significantly improved with liver transplantation (LT).^8,9^

Although living-donor LT (LDLT) for patients with PoPH has been previously performed,^10^ it is not typically an option in the United States because of a significant increase in intra- and posttransplant mortality,^11-13^ as well as because of ethical considerations for donors. Key factors in the decision-making process regarding eligibility for these patients include a PVR <240 dynes-s-cm^−5^, preserved right ventricular (RV) function and cardiac output (CO), and an adequate response to pulmonary vasomodulator therapy.^12^ Under these circumstances, a mPAP >35 mm Hg can be accepted, and LT can be safely performed.^14,15^

In 2021, the Penn Transplant Institute established a protocol for preoperative evaluation, optimization, and management of LT candidates with PoPH (Figure 1). Although the protocol had previously been successfully employed to care for recipients undergoing deceased donor LT, we describe its first use for a patient with severe PoPH undergoing LDLT.

**FIGURE 1.**
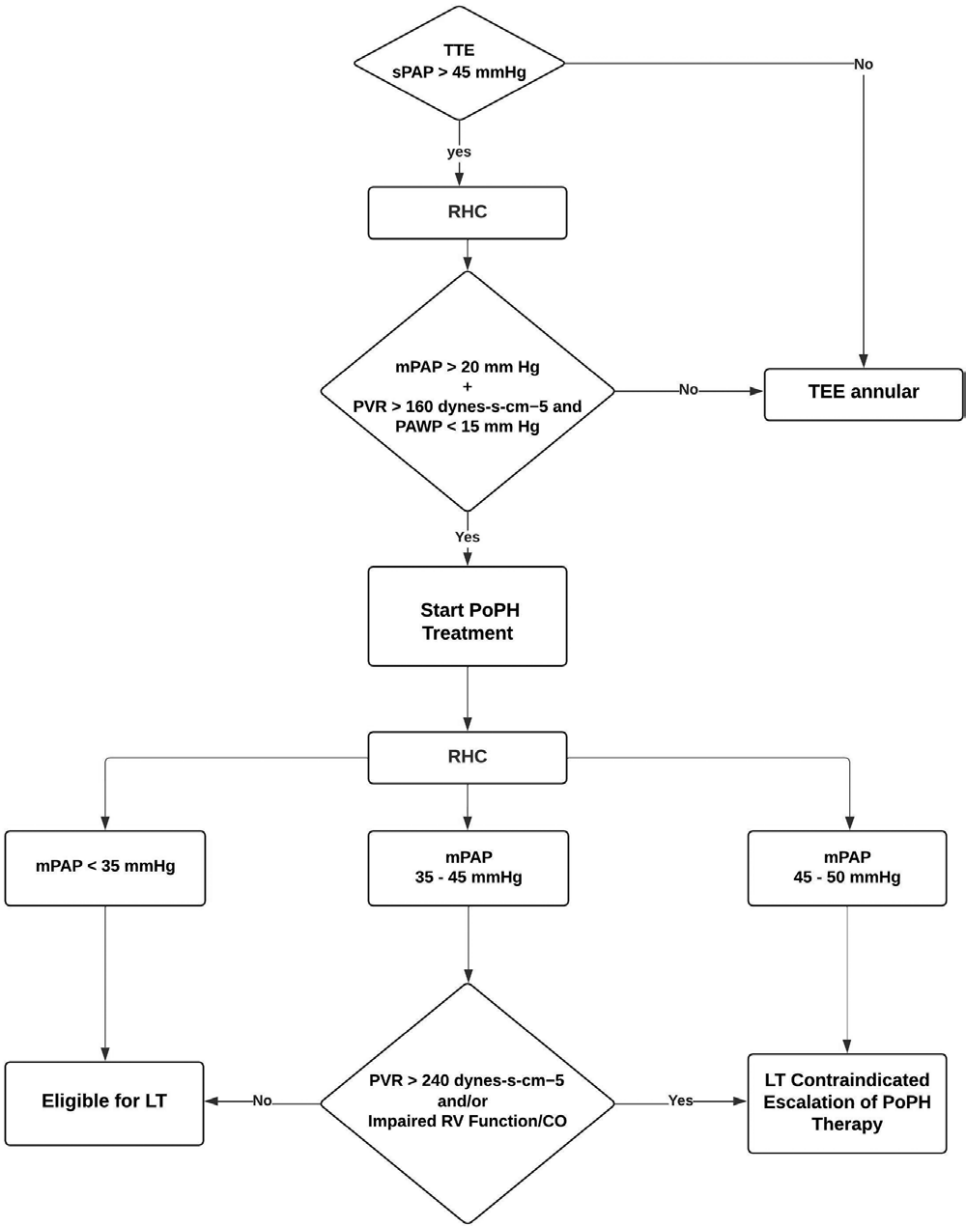
Penn Transplant Institute Guideline: portopulmonary hypertension. CO, cardiac output; LT, liver transplantation; mPAP, mean pulmonary artery pressure; PoPH, portopulmonary hypertension; PAWP pulmonary capillary wedge pressure; PVR, pulmonary vascular resistance; RHC, right heart catheterization; RV, right ventricle; sPAP, systolic pulmonary artery pressure; TTE, transesophageal echocardiography. Modified based on DuBrock.^3^

## CASE DESCRIPTION

### Preoperative Course

Our patient was a 51-y-old male, American Society of Anesthesiology Physical Status 4, with decompensated liver disease secondary to nodular regenerative hyperplasia. His Model for End-stage Liver Disease (MELD) was 17, with additional exception points for PoPH. The patient’s end-stage liver disease was complicated by portal hypertension, esophageal varices with several bleeding episodes, a history of a transjugular intrahepatic portosystemic shunt, ascites status post large volume paracentesis, hepatopulmonary syndrome, and PoPH.

**FIGURE 2.**
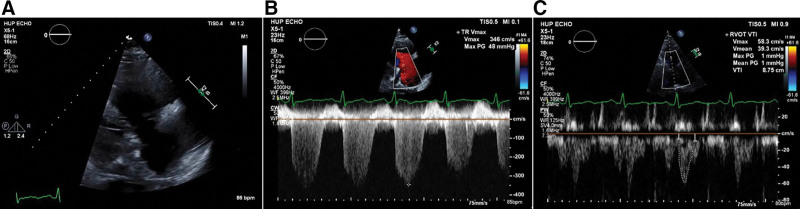
A–C, Transthoracic echocardiography 12 mo before liver transplantation. A moderate-to-severely dilated right ventricle with moderately decreased function, moderate-to-severe tricuspid regurgitation with pulmonary artery systolic pressure measuring 87 mm Hg. Mid-systolic notching of the right ventricular outflow track velocity time integral is present.

Twenty-three months before LT, the patient started to experience progressive hypoxemia and dyspnea on exertion and was referred to pulmonology medicine. Arterial blood gas demonstrated a partial pressure of oxygen of 49 mm Hg (oxygen saturation of 88%) on room air and he required 15 of oxygen liters per minute during his 6-min walk test. Transthoracic echocardiography (TTE) demonstrated normal right-sided filling pressures, cardiac index of 4.3 L/min/m^2^, and a low PVR (Table 1). A bubble test demonstrated no patent foramen ovale but the presence of an extracardiac shunt with the late passage of bubbles, confirming a diagnosis of hepatopulmonary syndrome. Twelve months before the transplant, the patient was admitted for variceal bleeding complicated by hypoxemia and volume overload. Right heart catheterization (RHC) at that time demonstrated mPAP 48 mm Hg, PCWP 8 mm Hg, and PVR 560 dynes-s-cm^−5^ with preserved CO (Table 1). TTE was notable for normal left ventricular (LV) function, moderate-to-severe RV and right atrial dilation, moderately decreased RV systolic function, moderate-to-severe tricuspid regurgitation (TR), mid-systolic notching of the RV outflow tract velocity time integral, and interatrial septal bowing to the left consistent with elevated right atrial pressure (Figure 2A–C, **Supplemental Video 1**). These data establish a diagnosis of PoPH. Symptomatic improvement followed diuresis and the addition of macitentan and sildenafil. Although his oxygen requirement decreased to 3 L/min, his transplant status was made inactive pending further optimization.

**TABLE 1.** Pulmonary and systemic hemodynamics on the waiting list and perioperatively

Date, Months before LT	HR	sBP/dBP, mm Hg	RAP, Mm Hg	sPA/dPA, mm Hg	mPA, mm Hg	PCWP, mm Hg	CO, L/min	CI, L/min/m^2^	PVR, dynes-s-cm^−5^	SVR, dynes	Medications
04/22, 20	89	136/64	6	28/10	21	17	9.4	4.3	34.3	698	
12/22, 12	68	113/73	14	71/37	48	8	5.7	2.6	560	1.011	
04/23, 8	60	106/61	11	68/26	40	15	8.5	4.0	232	640	Macitentan and sildenafil
07/23, 5	57	108/59	6	58/14	30	10	11.19	5.16	143.2	496	Macitentan and sildenafil
12/24, before LT	76	106/65	11	56/22	36	11	8.0	5.9	215	676	Macitentan and sildenafil
12/24, DOS	73	111/67	9	54/21	34	12	12.3	5.6	188	472	Macitentan and sildenafil
12/24, POD1	81	111/68	9	71/25	41	6	16.8	8.2	167	348	Epinephrine, norepinephrine, vasopressin, iNO

CI, cardiac index; CO, cardiac output; DOS, day of surgery; HR, heart rate; iNO, inhaled nitric oxide; mPA, mean pulmonary artery pressure; RAP, right atrial pressure; PCWP, pulmonary capillary wedge pressure; POD, postoperative day; PVR, pulmonary vascular resistance; sBP/dBP; systolic/diastolic blood pressure, sPA/dPA, systolic/diastolic pulmonary artery systolic pressure; SVR, systemic vascular resistance.

**FIGURE 3.**
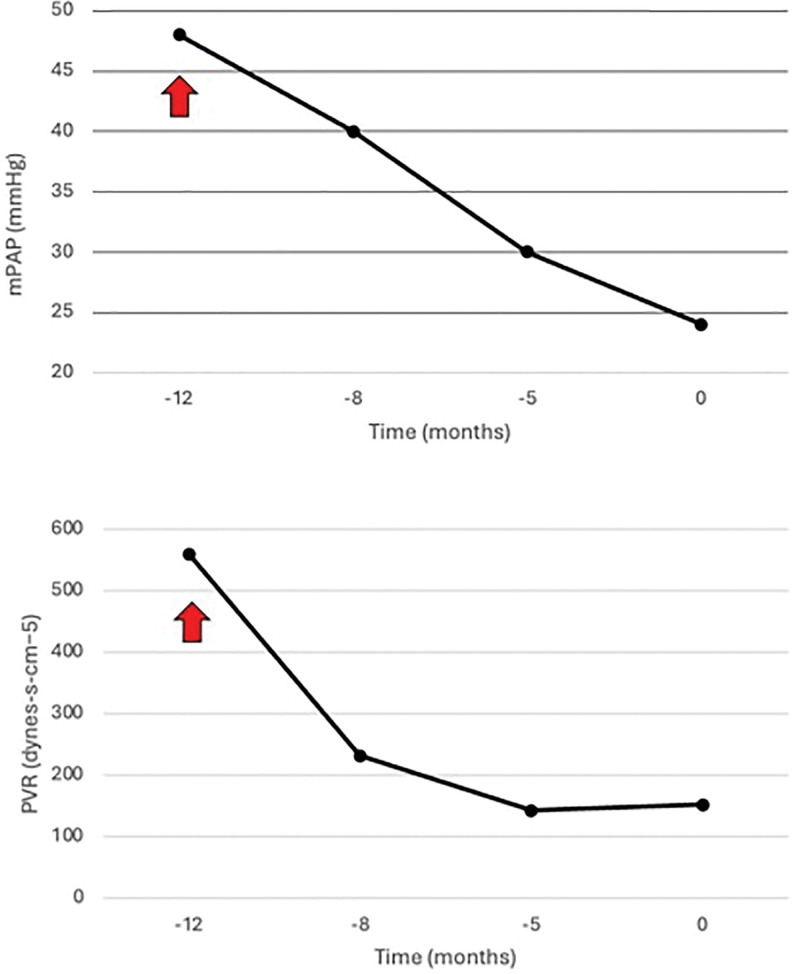
Change of mean pulmonary artery pressure and pulmonary resistance over 12 mo time period. Pretransplant mPAP and PVR as obtained by serial RHC. Time 0 denotes the month of LDLT. The upward arrow signifies initiation of oral pulmonary vasomodulator therapy (macitentan and sildenafil). LDLT, living-donor liver transplantation; mPAP, mean pulmonary artery pressure; PVR, pulmonary vascular resistance; RHC, right heart catheter.

After 7 mo of medical management with sildenafil, spironolactone, torsemide, and macitentan, RHC demonstrated an improvement of mPAP to 30 mm Hg and PVR of 143 dynes-s-cm^−5^ (Figure 3). TTE showed normal RV function with mild RA/RV dilation and mild TR. However, the patient was maintained on supplemental oxygen as needed during exertion. At this point, the patient met MELD exemption criteria for PoPH and was deemed to be an appropriate candidate for right lobe LDLT. A potentially suitable donor underwent evaluation for donation. The increased risk of LT in the setting of PoPH was extensively discussed with both donor and recipient, and they accepted the risk.

Preoperatively, a multidisciplinary meeting was held with transplant surgeons and anesthesiologists, pulmonary hypertension experts, and intensivists to formulate an overall perioperative plan using previously established institutional PoPH guidelines. Per the plan, the patient was preadmitted to the surgical intensive care unit (SICU) 1 d before surgery to facilitate placement of a continuous CO pulmonary artery catheter to document preoperative RHC values and confirm well-controlled PoPH. With an mPAP of 36 mm Hg, PVR of 215 dynes-s-cm^−5^, and CO of 8 L/min, the patient was deemed to be adequately optimized and he proceeded to the operating room the next day for transplantation.

### Intraoperative Course

After induction of general anesthesia and endotracheal intubation, a 16Fr Fem-Flex Duraflow cannula (Baxter, Irvine, CA) was placed percutaneously in the right jugular vein for the venovenous bypass (VVB), as is standard at our institution. VVB (between femoral, portal, and jugular veins) was used to ensure hemodynamic stability and decompression of the portal circulation. Bypass duration was 174 min.

A transesophageal echocardiography (TEE) probe and radial arterial line were placed for intraoperative cardiac monitoring. TTE findings were similar to preoperative evaluation and demonstrated severe left atrial dilatation, mild dilatation of the right atrium and RV, preserved LV (ejection fraction of 60%–65%), and RV function, and mild mitral and TR. Inhaled epoprostenol (Flolan) was delivered via the inspiratory limb of the ventilation circuit immediately after endotracheal intubation. Throughout the case, mild hyperventilation (with a target of PaCO_2_ 30–32 mm Hg) and a restrictive intravascular volume administration protocol (75 mL of fluid per hour) were used to reduce pulmonary pressure. The total case duration was 16.2 h, with a total cold ischemic time of 121 min.

The donor was a 44-y-old female who not biologically related to the recipient patient. The graft weighed 620 g, was of excellent quality, had a single artery, and no apparent steatosis. The graft-to-recipient weight ratio was 0.68.

**FIGURE 4.**
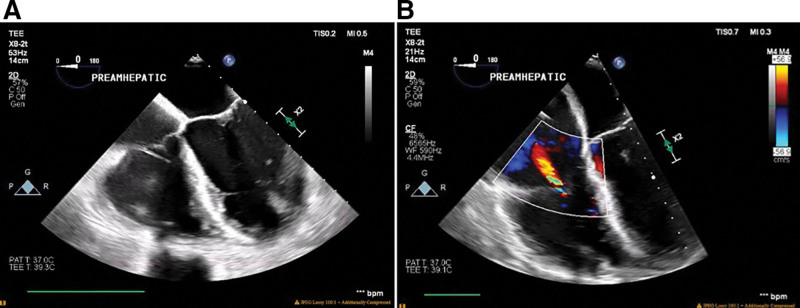
A and B, Intraoperative transesophageal echocardiography. RV dilation with preserved systolic function and mild TR on inhaled epoprostenol. Postreperfusion, RV function was hyperdynamic on epinephrine and vasopressin (not shown). RV, right ventricle; TR, tricuspid regurgitation.

**FIGURE 5.**
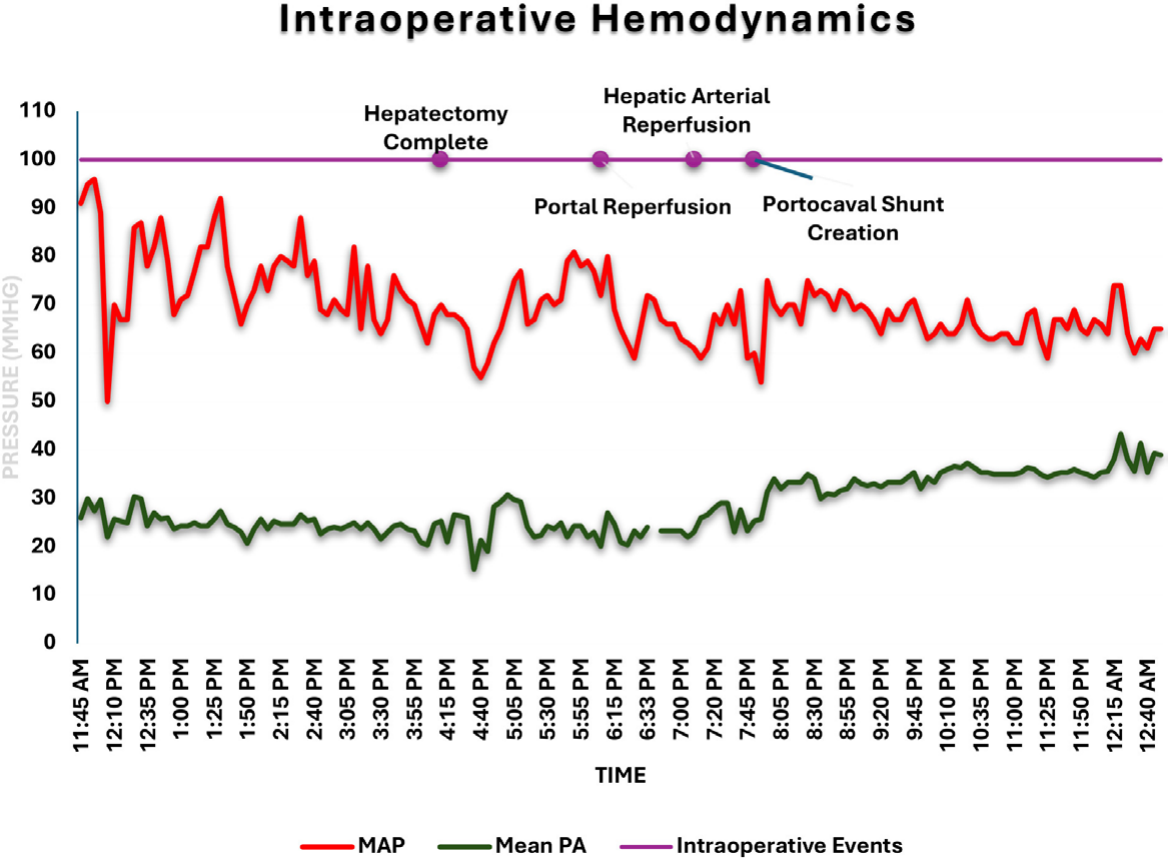
Intraoperative hemodynamics. Data illustrating MAP and mean PA pressure throughout prehepatic (procedure start to hepatectomy), anhepatic (hepatectomy to portal reperfusion), and neohepatic (postreperfusion) phases. The time of portocaval shunt creation was estimated to be 40 min post arterial reperfusion. MAP, mean arterial pressure; mean PA, mean pulmonary artery pressure.

TEE findings were significant for RV dilation with preserved systolic function and mild TR. Although cardiac function did not change significantly throughout the surgical procedure, immediately after hepatic graft reperfusion, deterioration of RV function was noted. RV dysfunction promptly resolved after epinephrine infusion (0.05 µg/kg/min) was started. Cardiac function remained stable and did not change significantly until the end of the procedure (Figure 4A and B; **Supplemental Video 2**). Intraoperative mean pulmonary pressures ranged from 30 to 40 mm Hg with occasional spikes to 50 mm Hg (Figure 5). Intermittent infusions of vasopressin and epinephrine were used for blood pressure and inotropic support.

After completion of the portal venous and hepatic arterial anastomoses, the liver became congested, likely because of a large degree of portal flow through a relatively small graft. This graft congestion was refractory to splenic artery ligation, so a portocaval shunt was created utilizing an external iliac artery graft. From a surgical standpoint, there were no major problems during the case, and graft congestion was improved before closing.

At the end of the case, the patient remained intubated and sedated and was transported to the SICU on epinephrine and vasopressin infusions and inhaled epoprostenol. Two units of packed red blood cells, 10 units of fresh–frozen plasma, 3 doses of platelets, and 3 L of crystalloid were administered during the case. Urine output was 1 L. Blood loss was not quantified.

In the immediate postoperative period, epoprostenol was transitioned to inhaled nitric oxide (iNO), and vasoactive infusions were titrated to a mean arterial pressure goal of 65 mm Hg. Caution was exercised with postoperative fluid resuscitation because of the patient’s significant RV dilation. Over the next few hours, the patient developed an increasing pressors requirement (with the addition of norepinephrine), decreased urine output, and a persistently elevated lactate level of 14 mmol/L. Using point-of-care ultrasound for interval assessments of RV function and volume status, 5% albumin was administered resulting in significant improvement in the overall hemodynamic profile.

A formal postoperative TTE was obtained on postoperative day (POD) zero, which demonstrated findings similar to those during surgery: significant RV dilatation with preserved RV and LV function. After consultation with the pulmonary hypertension team, the patient was immediately restarted on macitentan and later sildenafil after extubation on POD 2. Following our protocol, the patient was extubated to a high-flow nasal cannula (HFNC) with iNO. This therapy was started for both PoPH management as well as due to a concern for reduced hypoxic pulmonary vasoconstriction secondary to ongoing systemic vasodilatory treatment. Although iNO was able to be rapidly weaned, the patient remained in the SICU for several days because of a persistently elevated oxygen requirement despite large inspiratory volumes achieved on incentive spirometry and chest imaging demonstrating only mild pulmonary edema and atelectasis. This was felt to be due, at least in part, to a blunted hypoxic pulmonary vasoconstriction response resulting in shunt physiology. A large right pleural effusion was noted on POD 12, which was treated with thoracentesis, draining 2 L of fluid. Alternating between HFNC during the day and bilevel-positive airway pressure (BiPAP) at night, the patient was eventually weaned to a nasal cannula on POD 17 and was deemed safe to be transferred out of the SICU.

An RHC performed on POD 22 for persistent hypotension demonstrated an mPAP of 24 mm Hg, PVR 152 dynes-s-cm^−5^, CO 11.2 L/min, RA pressure 5 mm Hg, PCWP 9 mm Hg, and SVR 645 dynes-s-cm^−5^. With a normal PVR, sildenafil was discontinued, IV was fluid administered, and midodrine was stared (for low SVR).

The patient was discharged to acute rehabilitation on POD 34, and home on POD 41. He was weaned to room air 2 mo post LT. To date, he remains on midodrine, macitentan, and torsemide and has transitioned to tadalafil. TTE 4 mo postoperatively revealed mild to moderately RV dilation with borderline normal function, mild TR, and mild pulmonary arterial hypertension with an mPAP of 46 mm Hg. He reports symptomatic improvement, is exercising at home and in outpatient rehabilitation, and is back to work 3 d/wk.

## DISCUSSION

LT (and particularly LDLT) in the setting of significant PoPH is frequently considered to be contraindicated at many institutions. However, it has been demonstrated that early initiation of vasomodulatory therapy followed by LT can be associated with significantly improved survival.^10,16,17^ LDLT can confer significant advantages, including allowing patients to receive a superior-quality graft, particularly in subpopulations with a lower chance for deceased donor organ offers.

Over the last few years in the United States, approximately 600 LDLT were performed yearly (OPTN report). This number in 2020 was 401 and 282 in 2010. This is significantly lower compared with other countries using living donors as a main source of organs for LT. There are several reasons for this, but ethical considerations with LDLT are one of the most important in the United States. These ethical factors are in place to minimize the risk to donors who have no medical benefit from donation.^18^ Nizamuddin et al^18^ defined the main principles for a donor’s medical ethics, including *autonomy* (providing donors sufficient information), *nonmaleficence* (minimizing medical and psychosocial complications), *utility* (careful analysis of LDLT in patients with potentially inferior outcomes), *beneficence* (understanding motivations), and social *equity* (inclusion of recipient social criteria in consideration).

Our donor was very motivated and accepted a higher risk of graft failure in the recipient with PoPH. The recipient had a relatively low MELD score and his PoPH was well-controlled during LT. LDLT would offer surgery in stable conditions and could be scheduled during the daytime with resource optimization. The use of superior-quality organs (such as a living donor graft) likely significantly increases the probability of a successful outcome. This surgery should be also performed in a center that routinely performs LDLT (30–35 cases a year at our institution).

As previously described, a multidisciplinary guideline was created at our institution in 2021 for patients with PoPH. This guideline directs screening and risk stratification for patients with end-stage liver disease with concomitant PoPH as well as determines suitability for LT. All patients being considered for LT are screened preoperatively for PoPH with a TTE which is repeated yearly while they are actively listed for transplant. If a patient’s mPAP is >45 mm Hg without signs of volume overload, RHC should be performed to exclude PoPH. Patients with an mPAP >35 mmHg with PVR >160 dynes-s-cm^−5^ are referred to pulmonary hypertension specialists for vasomodulatory therapy which includes modulation of prostacyclin, NO, and endothelin pathways. It has been demonstrated that elevated PVR is associated with a significantly increased hazard of mortality and graft failure in patients with PoPH.^19^ The response of patients to vasomodulatory therapy initiated in the pretransplant stage plays a crucial role in decision-making regarding the eligibility of patients for LT.^20^ Current guidelines recommend the use of a combination of inhaled phosphodiesterase 5 inhibitors (PDE5i) and endothelin receptor antagonists (ERA) for low- and intermediate-risk cases with the addition of parenteral prostacyclin for high-risk cases.^21^ The goal of preoperative treatment is to achieve low-risk status and improve both pulmonary hemodynamics and patient symptoms/quality of life.^3^

Our patient had significantly increased mPAP and PVR. PDE5i and ERA were started 12 mo before LT with an excellent response (Figure 3). It has been demonstrated that the combination of these agents as dual oral therapy is much more effective in decreasing PVR than monotherapy.^8^ PDE5i has been used for the treatment of PoPH for >20 y. Several studies have demonstrated their effectiveness in decreasing pulmonary pressure and improving both RV function and eligibility for LT.^22-24^ There is significant experience with the use of ERA for managing PoPH. Although bosentan has been associated with potential hepatoxicity,^23,25^effectiveness without liver impairment has been demonstrated for both ambrisentan and macitentan. In a recent multicenter, randomized, double-blinded, placebo-controlled study, treatment with macitentan was associated with significantly decreased PVR and mPAP as well as improvement in CO.^26^

Per protocol, a patient will be eligible for LT if all their pulmonary hemodynamic parameters improve with treatment. At the time of organ offer, or on the evening before a planned LDLT, patients with PoPH are admitted to the SICU where a continuous CO pulmonary artery catheter is placed under local anesthesia. Relevant hemodynamic variables such as pulmonary artery systolic and diastolic pressures, mPAP, RAP, PCWP at end-expiration, CO, cardiac index, and PVR are measured and/or derived with the patient awake in the supine position. Systemic, noninvasive blood pressure and heart rate are also recorded. A multidisciplinary discussion ensues regarding the patient’s suitability for transplant. This includes representatives from LT anesthesia and surgery, critical care, and pulmonary medicine. Criteria for approval include PVR below 240 dynes-s-cm^−5^, mPAP <45 mm Hg, and preserved CO. Further discussion regarding perioperative optimization and management will also take place before proceeding to the operating room.

Despite significantly improved mPAP and PVR during preoperative treatment, further intraoperative management of our patient’s hemodynamics was necessary. In addition to mild hyperventilation and restrictive fluid management, inhaled epoprostenol was used throughout the surgery. Intraoperative parenteral epoprostenol administration for patients with PoPH has been previously described.^10,27^ Although it is used infrequently for this indication, inhaled epoprostenol helps avoid systemic vasodilatation and hemodynamic instability.

The decision was made preoperatively that this patient would remain intubated and transported to the SICU with inhaled epoprostenol with a plan to extubate to HFNC with continued inhaled pulmonary vasodilatory therapy. At our institution, we extubate approximately 70% of LT recipients in the operating room and nearly all of our LDLT recipients. In patients with PoPH, it is important to ensure that there is no residual anesthesia agents and that there is adequate analgesia before extubation. Any hypoxemia or hypercarbia could result in increased PVR and potentially lead to RV failure and graft congestion. In addition, atelectasis because of poor respiratory effort or splinting will not be tolerated in the setting of systemic pulmonary vasodilator therapy, which inhibits hypoxic pulmonary vasoconstriction leading to shunt physiology. The use of inhaled pulmonary vasodilators limits this effect on nonventilated areas of the lung and causes less systemic vasodilation.

Although VVB is routinely used for all LTs at our institution, it is particularly beneficial for managing patients with PoPH.^28,29^ The main cause of intraoperative mortality in patients with PoPH is a combination of acute and chronic RV failure because of a sudden increase in preload in combination with release of several active mediators from the hepatic graft during reperfusion causing a sudden increase in pulmonary arterial afterload.^30,31^ This can lead to cardiac failure and acute graft congestion, which is difficult to manage. VVB decreases pressure in the portal circulation, helps maintain hemodynamic stability, preserve coronary perfusion, and prevents acute increases in preload.

In conclusion, LDLT for patients with significant PoPH is not only feasible but likely a better option in comparison with deceased donation. LDLT usually provides a patient with a superior graft which can improve long-term survival. Early diagnosis and management of PoPH often results in normalization of pulmonary hemodynamics. Transplantation can then be performed as an elective procedure, including VVB. Developing and following a multidisciplinary protocol for the perioperative management of LDLT in patients with PoPH is essential to obtain an optimal outcome.
